# Do People Explicitly Make a Frame Choice Based on the Reference Point?

**DOI:** 10.3389/fpsyg.2018.02552

**Published:** 2018-12-17

**Authors:** Hidehito Honda, Masaru Shirasuna, Toshihiko Matsuka, Kazuhiro Ueda

**Affiliations:** ^1^Department of Psychology, Yasuda Women’s University, Hiroshima, Japan; ^2^Graduate School of Arts and Sciences, The University of Tokyo, Tokyo, Japan; ^3^Department of Cognitive and Information Science, Chiba University, Chiba, Japan

**Keywords:** framing effect, reference point hypothesis, priming effect, choice behavior, information leakage, frame choice

## Abstract

Previous studies have shown that when choosing one of two logically equivalent frames (e.g., “half full” or “half empty”), people tend to choose based on a reference point. For example, when the amount of water in a glass with 500 ml capacity was originally 0 ml (or 500 ml), and then increased (or decreased) to 250 ml, people tend to express the amount of water in the glass as “half full” (or “half empty”). In the present study, we examined whether participants explicitly made a frame choice based on the reference point. We conducted four behavioral experiments relating to frame choice tasks. Specifically, participants were presented with a story-based or prime-based reference point and then made a frame choice. Furthermore, participants provided their reasons for the choice. Our findings on frame choices and their reasons can be summarized as follows. First, when participants were presented with a story-based reference point, some of them reported that they made frame choices based on the reference point. Second, when a reference point was presented as a prime, participants’ frame choices were affected by this reference point. However, almost no participants reported that they made frame choices based on the reference point. These results indicate that the effect of reference points on frame choices is robust and that people do not always explicitly make frame choices based on the reference point.

## Introduction

In the research field of judgment and decision making, numerous researchers have examined how decision makers are affected by logically equivalent but different expressions in their decisions (e.g., Tversky and Kahneman, 1981; Kühberger, 1998; Levin et al., 1998; Soman, 2004; Keren, 2011).

Recently, several researchers have discussed how people choose a frame in order to express outcomes or situations (e.g., McKenzie and Nelson, 2003; Sher and McKenzie, 2006, 2008; Keren, 2007; Honda and Matsuka, 2014). McKenzie and his colleagues proposed a hypothesis on frame choice behaviors, called *the reference point hypothesis*, which states that in describing a fixed state of proportionate affairs, speakers are more likely to describe the proportion in terms of “X1” when X1 has increased relative to the reference point (the norm, or what one would have expected) than when X1 has decreased relative to the reference point (Sher and McKenzie, 2006, p. 471).

Imagine a glass with a capacity of 500 ml that contains 250 ml water. The reference point hypothesis predicts that people describe the glass as “half full” when the glass previously contained 0 ml (i.e., low reference point) more often than when the glass previously contained 500 ml (i.e., high reference point). In this situation, McKenzie and Nelson (2003) and Sher and McKenzie (2006) reported that participants’ frame choice behaviors are consistent with these predictions. The reference point hypothesis can widely predict people’s frame choice behaviors in conveying quantitative information (Moxey and Sanford, 1993a,b; Teigen and Karevold, 2005; Keren, 2007; Juanchich et al., 2010; Honda and Yamagishi, 2017).

The reference point hypothesis is mute about whether a respondent explicitly makes the choice of the frame based on the reference point. Indeed, previous studies have only examined whether frame choice is consistent with the prediction of the reference point hypothesis. Thus, the empirical question of whether people explicitly make frame choices based on the reference point remains unanswered.

We note another intriguing empirical question. Previous studies have manipulated a reference point (e.g., a change in the amount of water in a glass) using a cover story. In such an experimental setting, the reference point is “overt,” and the effect of the reference point in frame choice may be enhanced. That is, the effect of the reference point in frame choice may be unique in such experimental paradigm. Does the reference point affect the respondent’s frame choice when the reference point is not overt? Many studies have shown that reference points play an important role in various psychological processes of judgment and decision making (e.g., Kahneman and Tversky, 1979; Tversky and Kahneman, 1992; Heath et al., 1999; Allen et al., 2017). Given that reference points affect various aspects of psychological processes and peoples’ verbal behaviors are widely explained in terms of reference point-based frame choice, a reference point that is not as overt as that in the cover story may still affect the choice of frame.

According to these considerations, we examined whether people explicitly make frame choices based on the reference point. In order to examine this issue, we asked participants to provide reasons for their frame choices. If participants explicitly make frame choices based on the reference point, they would describe the reference point as the reason for their frame choice. In contrast, if participants’ descriptions did not mention anything about the reference point, then we could assume that participants did not explicitly focus on the reference point in their frame choice.

In the following sections, we report the results of four behavioral experiments. In Experiment 1, we conducted a frame choice task that was basically identical to that in previous studies. The reference point was presented using a cover story. In the subsequent Experiments 2, 3A, and 3B, we used a new experimental paradigm for presenting a reference point: a reference point was presented in a priming task. Through these four experiments, we examined the above empirical questions.

## Experiment 1

Experiment 1 was conducted as a preliminary study for the following experiments. We used the same experimental paradigm as in McKenzie and Nelson (2003). In their experimental procedure, participants were asked to choose one of two logically equivalent frames based on the cover story in which the information about the task-relevant reference point was provided. In addition, we asked participants to report the reason for their frame choice. As we discussed in the Introduction, in this experimental paradigm the participant may realize the reference point and make frame choices based on it, as it is readily available. That is, participants will report that they made frame choices based on the reference point. However, the empirical question remains as to whether this is true, and we examined this issue in Experiment 1.

### Methods

#### Participants and Experimental Design

Two hundred Japanese respondents (*M_age_* = 43.75, *SD_age_* = 8.69, *n_female_* = 92, *n_male_* = 108) participated in this experiment. For their participation they received a coupon that could be redeemed for online shopping in Japan. They were recruited via a website and randomly assigned to one of the two groups (*n* = 100 in each group, the low and high reference point groups). Thus, Experiment 1 was conducted with a between-participant design.

In the analogous study of McKenzie and Nelson (2003), effect sizes (*h*) between 0.41 and 1.37 were observed. To the best of our knowledge, there have been no studies that have conducted the task of McKenzie and Nelson (2003) EXPERIMENT 1 in Japanese. Thus, we took a conservative position on the effect size for this task. A power analysis indicated that a sample size of approximately 100 participants per condition was required for the study to have 80% power to detect the effect of *h* = 0.4. Based on this analysis, we set the number of participants in our study at 100.

#### Task, Materials, and Procedure

Participants made a frame choice and provided the reason for their choice. In the frame choice, we presented the following cover story, as provided by McKenzie and Nelson (2003).

“A glass with 500 ml capacity in front of you is filled with 0 ml water. You then leave the room briefly and come back in 10 min to find that the water is now at the 250 ml level. What is the most natural way to describe the glass now?”

Participants were asked to choose which frame was more natural, “The glass is half full” or “The glass is half empty.”^1^ This cover story was used for the low reference point group. For the high reference point group, the first sentence was “A glass with 500 ml capacity in front of you is filled with 500 ml water.” After the frame choice, participants provided their reasons for choice. In particular, the participants were asked, “Why did you think that the frame you chose was more natural?” and instructed to type in the reasons in a textbox. There were no limitations on the number of characters.

This experimental task was conducted through the internet (the other tasks were completely unrelated to the present task).

### Results and Discussion

**FIGURE 1:**
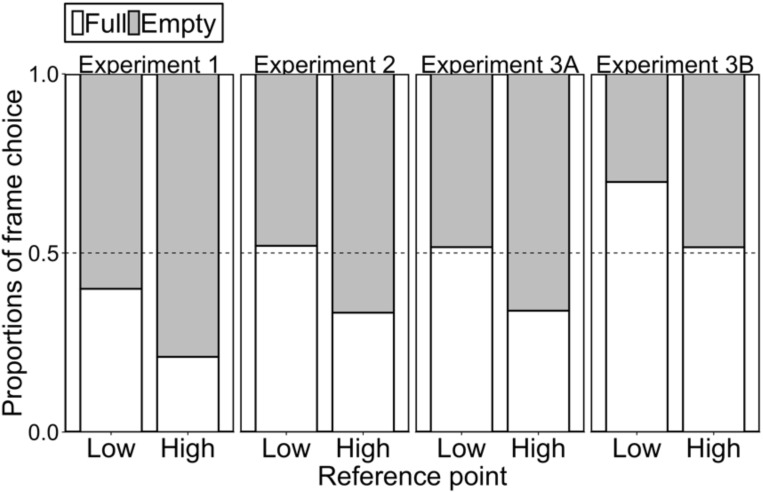
Proportions of frame choice (i.e., “half full” or “half empty”). The dotted line shows indifferent choice (no bias) between “full” and “empty” frames.

The left panel of Figure 1 shows the results of frame choice. Participants in the low reference point group chose the full frame more than those in the high reference point group did (40.0 and 21.0% for low and high reference point groups, respectively; *χ^2^*(1) = 7.64, *p* < 0.01, *h* = 0.42). This result corroborated the prediction of the reference point hypothesis.

As for the reason for their choice, we examined whether it was consistent with the reference point hypothesis for frame choices that were in accordance with its prediction (i.e., full frame choice for the low reference point group [*n* = 40] and empty frame choice for the high reference point group [*n* = 79]). We examined their reasons using the following procedure. Two independent evaluators who were unaware of the goal of the present research were first instructed about the reference point hypothesis. They checked whether each description was consistent with the reference point-based frame choice. Inconsistent evaluations were resolved between the evaluators.^2^ The first row of Table 1 shows the proportions of reference-point-based frame choice. We found that the proportions differed between the low and high reference point groups [*χ^2^*(1) = 14.15, *p* < 0.001, *h* = 0.79]. In total, 47.1% of the participants whose frame choices were consistent with the prediction from the reference point hypothesis stated that their choices were based on the reference point.

**Table 1:** Proportions of frame choice reasons for which participants stated a reference point.

	Low reference point	High reference point	Average
Experiment 1	0.725	0.342	0.471
Experiment 2	0	0	0
Experiment 3A	0	0	0
Experiment 3B	0.067	0.025	0.043


Although frame choice patterns were generally well predicted by the reference point hypothesis, the frame choice reasons varied. Thus, we analyzed the choice reasons for those participants who made a frame choice consistent with the reference point hypothesis. We focused on choice reasons other than the reference point-based frame choice^3^. For this, each choice reason was categorized by three independent evaluators (who were unaware of the present hypothesis) using the following procedure. In the first step, the first evaluator categorized the choice reasons after being presented with only the raw descriptions by participants (the evaluator did not know the participants’ group and the experiment number). This first evaluator was not given a list of categories. That is, the evaluator was asked to construct the categories as simply as possible. In this first step, four categories were constructed: linguistic nature (e.g., “chosen frame is a more natural expression”), physical nature (e.g., “emptiness is very salient”), no specific reason (e.g., “I don’t know, I chose intuitively”), and other reasons, which accounted for less than 5% of the total. In the second step, the other two evaluators categorized each choice reason into one of the four categories. Specifically, the second and third evaluators were presented with the raw choice reasons of participants (these evaluators were also unaware of the participants’ group and the experiment number); they then categorized each description into one of the four categories. Inconsistent categorizations were resolved by discussion between these two evaluators.^4^

The first row in Table 2 shows the results of this categorization in Experiment 1. Around 70% of the participants reported that their frame choices were based on linguistic nature reasons. This trend is understandable given the nature of the task. In Experiment 1, in which the reference point was presented by the cover story, the participants may have focused more on the linguistic nature for the two frames. As Experiment 1 was a preliminary study for the subsequent experiments, as we mentioned, we used these results as benchmark for comparison with results in Experiments 2, 3A, and 3B.

**Table 2:** Proportions of frame choice reasons other than the reference point-based choice.

Experiment	Linguistic nature		Physical nature		No reason		Others	
1	0.68	–	0.06	–	0.21	–	0.05	–
2	0.48	+	0.27	^∗∗^	0.21		0.04	
3A	0.35	^∗∗∗^	0.28	^∗∗^	0.14		0.23	^∗∗^
3B	0.33	^∗∗∗^	0.37	^∗∗∗^	0.17		0.13	


*Each number was calculated based on the participants whose frame choices were consistent with the prediction of the reference point hypothesis. +*p* < 0.1, ^∗^*p* < 0.05, ^∗∗^*p* < 0.01, ^∗∗∗^*p* < 0.001. These *p*-values indicate the results of multiple comparisons (Fisher’s exact test) for each proportion between Experiment 1 and each of the subsequent experiments (i.e., 2, 3A, or 3B). The *p*-values were adjusted using Bonferroni method for each experiment.*

Taken together, we found that when the reference point was presented through the cover story, as in previous studies (e.g., McKenzie and Nelson, 2003), around 50% of the participants who chose the frame consistent with the prediction of the reference point hypothesis reported that their choice was based on the reference point, indicating that around half of the participants explicitly made frame choices based on the reference point. We note that the proportions of participants who reported the effect of the reference point in frame choice differed between the low and high reference point groups (see Table 1). Our results imply that increase and decrease from the reference point differ psychologically and that such a difference affects the psychological processes of frame choice.

## Experiment 2

In Experiment 2, we examined whether a reference point affected the participant’s choice of frame even when the reference point was not overt. For this examination, we used the priming paradigm. Participants were presented with a reference point in a priming task. They then provided a frame choice and reported the reason for their choice.

We predicted that the reference point presented in the priming task would affect subsequent frame choice. However, they would not describe the reference point as a reason for their frame choice.

### Methods

#### Participants^5^ and Experimental Design

One hundred and fifty Japanese respondents (*M_age_* = 44.44, *SD_age_* = 8.28, *n_female_* = 74, *n_male_* = 76) participated in this experiment. They were recruited via a website and randomly assigned to one of the two groups (*n* = 75 in each reference point group). As in Experiment 1, we conducted the task using a between-participant design.

Since this was the first study to examine the effect of the priming task on the subsequent frame choice task, it might be difficult to set a clear criterion for the effect size to determine the sample size. We set the sample size as follows. Based on the findings of McKenzie and Nelson (2003) and Experiment 1, we assumed that a reasonable lowest effect size for the frame choice task would be 0.4. Then, in a general sense, we believed that medium effect size (*h* = 0.5, Cohen, 1988) would be a reasonable benchmark for determining the sample size for the second experiment. A power analysis indicated that a sample size of approximately 75 participants per condition was required for the study to have 80% power to detect such an effect (*h* = 0.4–0.5). Accordingly, we set the number of participants in our study at 75.

#### Task, Materials, and Procedure

**FIGURE 2:**
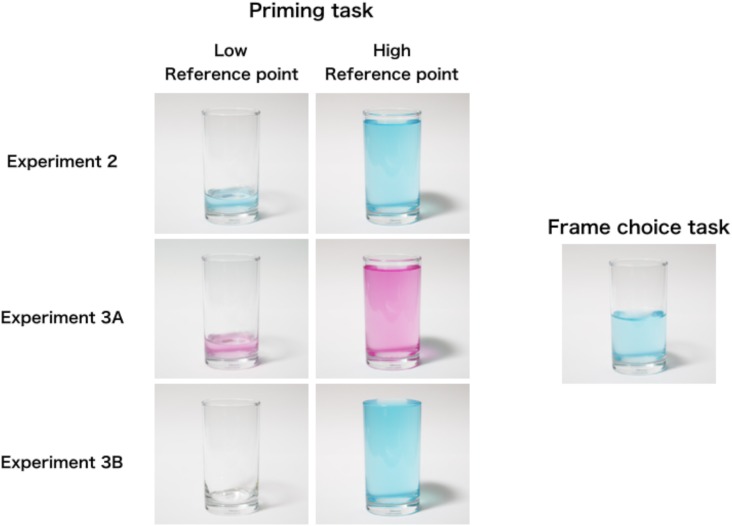
Pictures presented in Experiments 2, 3A, and 3B.

We conducted three tasks: a priming task, a frame choice task, and a reason description task. In the priming task, participants were presented with one of the two pictures (i.e., they were presented with a low or high reference point picture; see Figure 2) and provided an estimate of how much water was in the glass. For this task, participants were presented with the following instruction: “The glass with 500 ml capacity contains some water. Please estimate how much water the glass has.” The picture was presented during the estimation. After this (participants were asked to press the “Next” button when they finished the estimation), the picture disappeared and participants were presented with another picture of the glass (the picture for the frame choice task, see Figure 2) and made a choice of frame that naturally described the amount of water. In this task, we gave the following instruction: “Here, a glass with a 500 ml capacity in front of you is filled with 250 ml of water as in the picture. What is the most natural way to describe the glass?” Participants chose one of the two frames: “The glass is half full” or “The glass is half empty.” The instruction did not state anything about the relationship between the two pictures (i.e., the pictures presented in the priming and frame choice tasks). After the frame choice, participants provided the reason for their choices.

As in Experiment 1, this was an experimental task conducted through the internet (the other tasks were completely unrelated to the present task).

### Results and Discussion

First, we checked whether the manipulation of the priming task worked as we had expected. The means of the estimates for the amount of water were 87.6 (*SD* = 73.7) and 457.1 (*SD* = 65.6) for the low and high reference points, respectively. These estimates significantly differed [*t*(148) = 32.5, *p* < 0.001, *d* = 5.30]. Thus, we obtained the expected estimates for the two groups.

The middle left panel of Figure 1 shows the result of frame choice. Participants in the low reference point group chose the full frame more than those in the high reference point group (52.0 and 33.3% for low and high reference point groups, respectively; *χ^2^*(1) = 4.61, *p* < 0.05, *h* = 0.38), showing that choice pattern was consistent with the prediction of the reference point hypothesis. Thus, we found that the reference point presented in the priming task affected the frame choice.

As in Experiment 1, we examined whether the reason for choice was based on the reference point for frame choices that were consistent with the reference point hypothesis (*n* = 39 and 50 for the low and high reference point groups, respectively). The second row of Table 1 shows the proportions of the reference-point-based frame choice reasons. It was found that no participants stated that their choices were based on the prime, suggesting that they did not explicitly make frame choices based on the reference point (i.e., prime).

We analyzed the contents of choice reasons other than the reference point-based frame choice for those participants who made a frame choice consistent with the reference point hypothesis. The second row of Table 2 shows the results of the categorization in Experiment 2. Compared with those in Experiment 1, the proportion of participants who reported “linguistic nature” reduced (*p* = 0.05), whereas more participants reported “physical nature” (*p* = 0.005). In Experiment 2, the reference point was presented by a picture (see Figure 2), and the participants may have focused more on the physical nature of the contents of the glass.

In the priming task, participants were presented with one of the pictures of the glass that contained water, then provided an estimate of the amount of water. We note that the experimental procedure in Experiment 2 might have produced a strong effect of the reference point (i.e., prime) for the following two reasons. First, since the color of the liquid in the prime task was the same as in the frame choice task (see Figure 2), the participants might have regarded the prime (the picture of the glass) as equivalent to the picture in the frame choice task and thought that the amount of water “increased” or “decreased.” Thus, although no participants reported the reference-point-based choice, the presented pictures might have worked as a “relevant” reference point. Second, the number that participants provided as estimates might have worked as an anchor and thereby affected frame choice. Previous studies have shown that a precedent numerical stimulus, called an *anchor*, significantly affects subsequent numerical estimation (e.g., Tversky and Kahneman, 1974). The priming task in Experiment 2 (i.e., the numerical estimation of the amount of water in a glass) might have affected the subsequent frame choice because of the preceding numerical estimate. When a participant provided 80 (or 450) ml as an estimate, this value might have become an “anchor,” and comparison between the “anchor” and 250 ml (i.e., the amount of water in the frame choice task) might have generated a subjective sense of increase (or decrease) in the amount of water, which might have affected the frame choice. Conversely, what if the participants do not make numerical estimations for the amount of water in the glass before making their frame choice? If the estimation of the amount of water plays the role of “anchor” and affects subsequent frame choice, then it follows that the unique procedure of providing a numerical estimate in the priming task might have produced the strong effect of the prime. That is, when participants are not asked to make a numerical estimation (i.e., providing “number”) for the prime, the prime may not affect subsequent frame choice.

We shall discuss these two issues in Experiments 3A (addressing the first issue of equivalence of liquid color) and 3B (addressing the second issue on the effect of numerical estimation).

## Experiment 3A

### Methods

#### Participants and Experimental Design

Two hundred and forty-three Japanese respondents (*M_age_* = 46.45, *SD_age_* = 8.19, *n_female_* = 56, *n_male_* = 187) participated in this experiment. They were recruited via a website and randomly assigned to one of the two groups (*n* = 122 and 121 in the low and high reference point groups, respectively). As in the former experiments, we conducted the task under a between-participant design.

In Experiment 2, we observed the effect size in frame choice as *h* = 0.38. Thus, we assumed that setting 0.35 < *h* < 0.4 as the benchmark of the effect size in the priming task for frame choice would be reasonable. A power analysis indicated that a sample size of approximately 100–130 participants for each condition was required for the study to have 80% power to detect such an effect, upon which basis we recruited participants.

#### Task, Materials, and Procedure

The task, materials, and procedure were basically the same as in Experiment 2 with the exception of the stimuli in the priming task. Participants were presented with one of the pictures in which the color of the liquid differed from that in the frame choice task (see Figure 2). By presenting a picture with liquid of a different color, we excluded the possibility that participants would regard the prime as equivalent to the glass for the frame choice task.

### Results and Discussion

First, as in Experiment 2, we checked whether the manipulation of the priming task worked as we had expected. The means of the estimates of the amount of water were 95.8 (*SD* = 83.5) and 462.2 (*SD* = 79.6) for the low and high reference points, respectively. These estimates significantly differed [*t*(241) = 35.0, *p* < 0.001, *d* = 4.49]. Thus, we obtained the expected estimates for the two groups.

The middle right panel of Figure 1 shows the result of frame choice. Participants in the low reference point group chose the full frame more than those in the high reference point group (51.6 and 33.9% for low and high reference point groups, respectively; *χ^2^*(1) = 7.11, *p* < 0.01, *h* = 0.36), showing that choice pattern was consistent with the prediction of the reference point hypothesis.

As in the former experiments, we examined whether the reason for choice was based on the reference point for frame choices that were consistent with the reference point hypothesis (*n* = 63 and 80 for the low and high reference point groups, respectively). The third row of Table 1 shows the proportions of the reasons for the reference-point-based frame choice. No participants stated that their choices were based on the prime.

Finally, we analyzed the contents of choice reasons other than the reference point-based frame choice for those participants who made a frame choice consistent with the reference point hypothesis. The third row of Table 2 presents the results of the categorization in Experiment 3A. Compared with those in Experiment 1, the proportion of participants who reported “linguistic nature” reduced (*p* < 0.001), whereas more participants reported “physical nature” (*p* = 0.001). In addition, the proportion of participants who reported “others” significantly increased compared with those in Experiment 1 (*p* = 0.005). These results were generally consistent with those in Experiment 2.

In sum, the findings in Experiment 3A were highly analogous to those in Experiment 2. These results indicate that frame choices are affected by the reference point presented in the priming task.

## Experiment 3B

### Methods

#### Participants and Experimental Design

Two hundred and forty Japanese respondents (*M_age_* = 46.85, *SD_age_* = 8.23, *n_female_* = 61, *n_male_* = 179) participated in this experiment. They were recruited via a website and randomly assigned to one of the two groups (*n* = 116 and 124 in the low and high reference point groups, respectively). As in former experiments, we conducted the task as between-participant design^6^.

#### Task, Materials, and Procedure

The task, materials, and procedure were basically the same as in Experiments 2 and 3A with the exception of the priming task. In Experiment 3B, participants were presented with one of the pictures (see Figure 2) and asked how much they liked it. They expressed their preferences with “I like,” “Neither like nor dislike,” or “I don’t like.” Thus, unlike the procedure in Experiments 2 and 3A, the participants did not provide a numerical estimate, but only their preference for the picture.

### Results and Discussion

First, we examined the preference for pictures between the two groups. The proportions of preferences for “I like,” “Neither like nor dislike,” and “I don’t like” were 0.095, 0.776, and 0.129 for the low reference point group, and 0.274, 0.613, and 0.113 for the high reference point group, respectively. There was a significant difference in preference for pictures between the two groups [*χ^2^*(2) = 12.72, *p* < 0.01, *φ_c_* = 0.16], showing that participants in the high reference point group preferred the picture more than those in the low reference point group. This result was unexpected.

The right panel of Figure 1 shows the result of frame choice. Participants in the low reference point group chose the full frame more than those in the high reference point group [69.8 and 51.6% for low and high reference point groups, respectively; *χ^2^*(1) = 7.57, *p* < 0.01, *h* = 0.38]. This result was consistent with the prediction of the reference point hypothesis.^7^

As in the previous experiments, we examined whether the reason for the participant’s choice was based on the reference point for frame choices that were consistent with the reference point hypothesis (*n* = 81 and 60 for the low and high reference point groups, respectively). The forth row of Table 1 shows the proportions of the reference point-based frame choice. Six participants (two from the low and four from the high reference point group; in total 4.3%) reported that their choices were based on the presented picture in the priming task (e.g., “In comparison with the picture presented before, the amount of water increased. The amount of water has changed into the full direction, so it means that the content of the glass has become full”). However, most participants did not state the effect of the prime on their frame choice.

Finally, we analyzed the contents of choice reasons other than the reference point-based frame choice for those participants who made a frame choice consistent with the reference point hypothesis. The fourth row of Table 2 lists the results of the categorization in Experiment 3B. Compared with those in Experiment 1, the proportion of participants who reported “linguistic nature” reduced (*p* < 0.001), and more participants reported “physical nature” (*p* < 0.001). These results were generally consistent with those in experiments using the priming paradigm (i.e., Experiments 2 and 3A).

Taken as a whole, the findings of Experiment 3B were highly analogous to those of Experiment 2. These results indicate that frame choices are affected by the reference point presented as a prime, and almost all the participants did not explicitly make frame choices based on the reference point. Note that participants in Experiment 3B did not provide numerical estimates in the priming task. Thus, these results indicate that the effect of prime-based reference point was not generated by the unique procedure of the priming task (i.e., numerical estimation).

## General Discussion

We conducted a total of four behavioral experiments to examine the issue: do people explicitly make a frame choice based on the reference point? Our findings are summarized as follows: in the experimental paradigm, as in previous studies, in which a reference point was presented based on a cover story, around half of the participants reported that their frame choices were based on the reference point (Experiment 1). In contrast, when a reference point was presented as a prime, it also affected participants’ frame choices, although almost all of the participants did not report an effect of the reference point (Experiments 2, 3A, and 3B). The present findings have two important implications. First, the effect of reference points on frame choice is highly robust. Second, a respondent does not always explicitly make a frame choice based on the reference point.

We note that the present results suggest some language differences. In their EXPERIMENT 1, McKenzie and Nelson (2003) found that English speakers tended to prefer “full” frame to “empty” frame (72% vs. 28%). However, in the present study (i.e., with Japanese speaker participants), participants preferred the “empty” frame in general (45% for “full” frame vs. 55% for “empty” frame in the four experiments). At this point, we do not have any hypotheses about this language difference. Although the general preference for “full” or “empty” may vary across languages, it is noteworthy that the reference point hypothesis, which was originally proposed based on English speakers’ habits (McKenzie and Nelson, 2003), has been repeatedly corroborated by experiments in Japanese.

If a person makes a frame choice inconsistent with the prediction of the reference point hypothesis and her/his frame choice reason is based on the reference point, it follows that her/his frame choice violates the reference point hypothesis (i.e., s/he focuses on the decrease of the proportion). We analyzed the choice reasons for participants whose frame choices were inconsistent with the reference point hypothesis in the same way as reported in the experiment sections.^8^ In Experiment 1, 3 out of 60 participants in the low reference point group reported that they chose the empty frame based on the reference point (e.g., “because the amount of water increased from the emptiness”), and 1 of 20 participants in the high reference point group chose the full frame based on the reference point (e.g., “Originally, the amount was full and the amount decreased to half.”). Thus, in total, 5% of the participants reported choice reasons that were opposite from the prediction of the reference point hypothesis. In Experiments 2, 3A, and 3B, no participants reported an opposite choice reason. Altogether, few participants reported choice reasons that were inconsistent with the reference point hypothesis.

Given that most participants reported many kinds of choice reasons rather than reference points (see Table 2) in Experiments 2, 3A, and 3B, frame choice reasons might have been misattributed. In the discussion in Payne et al. (2016), the psychological processes of the priming effect were explained as follows: a prime makes a person think about the target behavior (e.g., in the present study, frame choice based on a reference point [i.e., prime]) and s/he mistakenly thinks that such psychological content is self-generated. Actually, as psychological processes are not always difficult to monitor (e.g., Nisbett and Wilson, 1977; Zajonc, 1980), a variety of *post hoc* reasons for the frame choice may have been generated.

Previous studies have shown that the selected frames can become important linguistic cues from which listeners can infer background information, such as situational shifts (e.g., shifts in the amount of water) or speakers’ trust (Sher and McKenzie, 2006; Keren, 2007). Such effective inferences can be made because listeners can effectively make inferences based on the speakers’ conversational tendencies (e.g., when speakers prefer “full” frames). Our findings suggest that people do not always explicitly follow the reference point hypothesis, as explicit frame choice reason can vary (see Table 2), although frame choice patterns are generally consistent with the reference point hypothesis. Especially, frame choice patterns were consistent with the prediction of the reference point hypothesis in Experiments 2, 3A, and 3B, in which the experimental procedure was manipulated such that the reference point did not become overt. Therefore, the shift from the reference point, which is one of the most important essences in the reference point hypothesis, may not be important to a speaker and s/he may make a frame choice with reasons other than the reference point. Future research could examine what information listeners explicitly read from the presented frame and how effective communication can be achieved between a speaker and a listener.

Finally, we shall point out an issue about the effect of primes on frame choice. In the present study, frame choice task was carried out just after the priming task was conducted. Intuitively, the effect of the prime seems transient. That is, when there is a time lag between the priming and frame choice tasks, the effect of the prime may disappear. In future research, it will be necessary to examine how long the effect of the prime-based reference point on frame choice persists.

## Ethics Statement

Tasks in the three experiments were approved by the Ethics Committee of Graduate School of Arts and Sciences at the University of Tokyo (Approval No. 380). All of the participants provided their web-based informed consent instead of written consent.

## Author Contributions

All authors developed the study concept and contributed to the study design. HH: performed the data collection. HH and MS: performed the testing and data analysis. HH wrote the manuscript, with feedback from MS, TM, and KU.

## Conflict of Interest Statement

The authors declare that the research was conducted in the absence of any commercial or financial relationships that could be construed as a potential conflict of interest.

## Footnotes

1 The original Japanese expressions were “*Gurasu no naka ha hanbun ippai dearu*” (The glass is half full) and “*Gurasu no naka ha hanbun kara dearu*” (The glass is half empty). The same expressions were used in Experiments 2, 3A, and 3B.

2 This procedure for the examination of reasons for choice was the same in Experiments 2, 3A, and 3B. Accordance rates were 97.4, 100, 100, and 97.9% for Experiments 1, 2, 3A, and 3B, respectively.

3 Although we report the analyses of choice reasons that were other than the reference point-based frame choice in each experiment, we actually conducted these analyses after all the four experiments were completed. Furthermore, the categorization of choice reasons was conducted by pooling all the choice reasons for Experiments 1, 2, 3A, and 3B.

4 Accordance rates were 96.8, 92.1, 93.0, and 93.0% for Experiments 1, 2, 3A, and 3B, respectively.

5 In Experiments 2, 3A, and 3B, when participants completed the tasks, they were asked about whether they had formerly experienced the analogous tasks of the frame choice. For the participants who had experienced the analogous tasks, we excluded their data. With this criterion, we deleted two and three participants from the data in Experiments 3A and 3B, respectively.

6 The policy for determining sample size was the same as in Experiment 3A.

7 As described above, the preferences for pictures differed between the two groups. Thus, the frame choice result may have been derived from the preference for the pictures presented in the priming task. We examined whether the difference in preference for the picture was related to the frame choice. The proportions for the “full” frame choice were 0.422, 0.386, and 0.414 for participants whose preferences were “I like” (*n* = 45), “Neither like nor dislike” (*n* = 168), and “I don’t like” (*n* = 29), respectively. Since there was no significant difference in the proportion for the “full” frame choice among the participants who showed different preferences [*χ^2^*(2) = 0.244, *p* = 0.89, *φ_c_* = 0.02], we can assume that preference for the picture was not significantly related to the frame choice.

8 Accordance rates of reason dichotomization between two evaluators were all 100% for Experiments 1, 2, 3A, and 3B.
